# Cell‐based therapies for the treatment of rheumatoid arthritis

**DOI:** 10.1002/iid3.1091

**Published:** 2023-11-22

**Authors:** Maryam Zare Moghaddam, Mohammad Javad Mousavi, Somayeh Ghotloo

**Affiliations:** ^1^ Department of Immunology Shahid Sadoughi University of Medical Sciences Yazd Iran; ^2^ Department of Hematology Faculty of Allied Medicine, Bushehr University of Medical Sciences Bushehr Iran; ^3^ Autoimmune Diseases Research Center Kashan University of Medical Sciences Kashan Iran; ^4^ Department of Clinical Laboratory Sciences Kashan University of Medical Sciences Kashan Iran

**Keywords:** none‐stem cell, rheumatoid arthritis, stem cell, therapy

## Abstract

Autoimmune diseases, including rheumatoid arthritis that is the most prevalent rheumatic autoimmune disorder, affect autologous connective tissues caused by the breakdown of the self‐tolerance mechanisms of the immune system. During the last two decades, cell‐based therapy, including stem cells and none‐stem cells has been increasingly considered as a therapeutic option in various diseases. This is partly due to the unique properties of stem cells that divide and differentiate from the specialized cells in the damaged tissue. Moreover, stem cells and none‐stem cells, impose immunomodulatory properties affecting the diseases caused by immunological abnormalities such as rheumatic autoimmune disorders. In the present review, the efficacy of cell‐based therapy with four main types of stem cells, including mesenchymal stem cells, hematopoietic stem cells, embryonic stem cells, and human amniotic membrane cells, as well as none‐stem cells, including regulatory T cells, chimeric antigen receptor T cells, and tolerogenic dendritic cells will be evaluated. Moreover, other related issues, including safety, changes in immunological parameters, suitable choice of stem cell and none‐stem cell origin, conditioning regimen, limitations, and complications will be discussed.

## Highlights

- Cell‐based therapy for rheumatoid arthritis was evaluated.
- Efficacies of stem cells and non‐stem cell therapies were evaluated.
- Issues, including conditioning regimen, limitations, and complications, were discussed.

## Graphical abstract

Stem cell therapy for systemic sclerosis was evaluated. The efficacy of mesenchymal and hematopoietic stem cells was evaluated. Issues, including conditioning regimen, limitations, and complications, were discussed

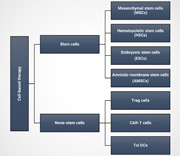

## 1 STEM CELL THERAPY (SCT) IN RHEUMATOID ARTHRITIS (RA)

RA is a chronic inflammatory autoimmune disease of joints. In RA, inflammation stimulates the formation of pannus tissue leading to cartilage and bone erosion. The disease affects 1% of people throughout the world and is more prevalent in females than males. Although the etiology of RA has not been fully discovered, but rheumatoid factor (RF) and an imbalance between T helper (Th)1/Th2 ratio as well as Th17/regulatory T cells (Treg) are associated with the disease pathogenesis resulting in a chronic inflammation. While inflammatory cytokines such as tumor necrosis factor‐α (TNF‐α), interleukin (IL)‐1, and IL‐17 act as pathogenic cytokines in RA, anti‐inflammatory cytokines such as IL‐10, and transforming growth factor‐β (TGF‐β) are ameliorating cytokines. In addition to autoreactive immune cells migrating into the synovium of RA patients, altered fibroblast‐like synoviocytes (FLSs) play crucial roles in the damage to cartilage and bone.^1^

**Figure 1:**
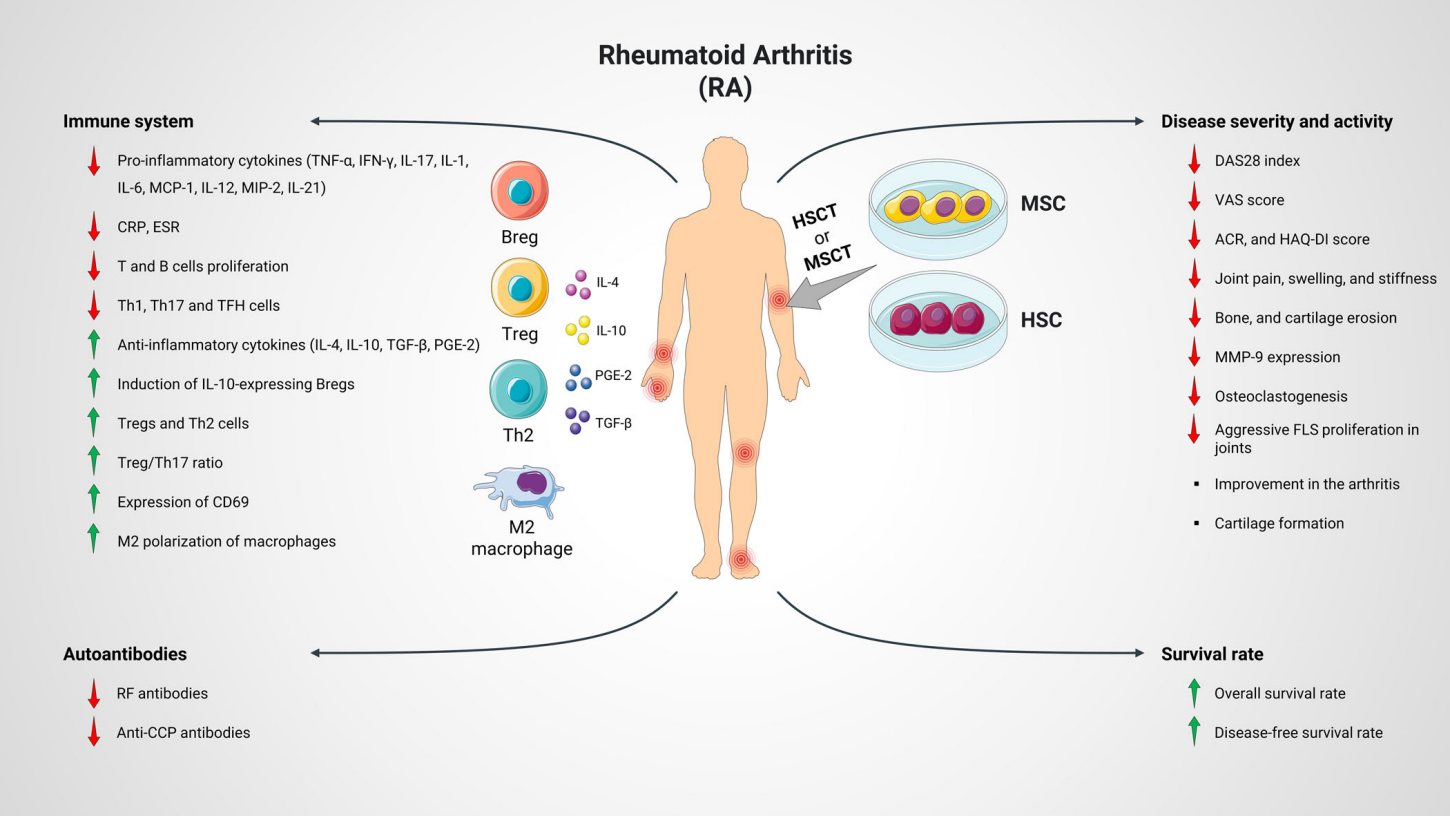
Effect of MSC/HSC therapy on the disease severity, activity, survival rate, and immune response. MSCT/HSCT leads to an improvement in the disease severity and activity. Moreover, an improvement in the survival rate was detected. MSCT/HSCT improved immunological parameters toward protective immune response. ACR, American College of Rheumatology; DAS28, disease activity score 28; FLS, fibroblast‐like synoviocytes; HAQ‐DI, Health Assessment Questionnaire‐Disability Index; HSC, hematopoietic stem cells, HSCT, hematopoietic stem cell transplantation; IFN‐γ, interferon‐γ; IL‐10, interleukin 10; MCP‐1, monocyte chemotactic protein‐1; MIP‐2, macrophage inflammatory protein‐2; MMP‐9, matrix metallopeptidase 9; MSC, mesenchymal stem cell; MSCT, mesenchymal stem cell transplantation; PGE2, prostaglandin E2; RF, rheumatoid factor; TGF‐β, tumor growth factor‐β; Th, T helper; TNF‐α, tumor necrosis factor‐α; Treg, regulatory T cell; VAS, visual analog scale.

In the last decade, conventional therapy, including disease‐modifying antirheumatic drugs (DMARDs) such as methotrexate (MTX), and biological therapy, including cytokine therapy such as TNF‐α inhibitors (TNF‐αi) have been used for the treatment of RA patients.^2^ Patients that do not respond to the conventional and biological treatments could be considered as candidates for SCT.^3^ Results of SCTs, including mesenchymal stem cells transplantation (MSCT), and hematopoietic stem cells transplantation (HSCT) in RA animal models or human patients will be reviewed in the next sections (Tables 1 and 2) (Figure 1).

**Table 1:** Results of stem cell therapy (SCT) in animal models of rheumatoid arthritis (RA).

Number	Type of stem cell	Result of SCT	Animal model/human	References
1	Allogeneic MSCs	Prevention of erosion in bone, and cartilage Increased Tregs	CIA mice model	[4, 5]
2	Human UC‐MSCs	Improvement in the disease Reduced expression of TNF‐α, IL‐6, MCP‐1 Increased expression of IL‐10 Reduced differentiation of Th1 and Th17 Induction of Tregs	CIA mice model	[6]
3	UC‐MSCs	Reduced frequency and stimulatory function of TFH Downregulation of inflammatory Response	CIA mice model	[7]
4	AD‐MSCs	Prevention of bone destruction Inhibition of osteoclastogenesis Induction of immune tolerance	CIA mice model	[8]
5	AD‐MSCs	Reduction of autoimmune response Lower damage to the cartilage Improvement in the arthritis	CIA mice model	[8, 9]
6	BM‐MSCs	No prevention of the disease progression No inhibition of T cells proliferation	CIA mice model	[10]
7	Engineered IL‐10‐MSCs	Inhibition of the disease initiation Reduced severity of arthritis Inhibition of T cells proliferation in response to CII A and decrease in IL‐6 and anti‐CII antibody	CIA mice model	[11]
8	Engineered Brachyury‐MSCs	Cartilage formation Prevention of cartilage destruction	SCID mice	[12, 13]
9	Engineered TGF‐β‐MSCs	Amelioration in arthritis Inhibition of osteoclast differentiation Balance in Tregs/Th17 ratio Reduction of proinflammatory cytokines	CIA mice model	[14]
10	Engineered xenogeneic CTLA4‐hAD‐MSCs	Decrease in severity of the cartilage injury Reduction in the CTX‐II level	CIA mice model	[15, 16]
11	Engineered CTLA4Ig‐hAD‐MSCs	Decrease in arthritis score Decreased serum level of IL‐12 Decrease of MIP‐2 in knee‐joint extract Decreased expression of T‐bet and GATA‐3 in splenocytes Increased ration of CD4 + CD25 + FoxP3 + Treg/CD4 + CD25 + RORγt + Th17 cells	CIA mice model In vitro studies	[16]
12	Combination of MSCs and IL‐4	Alleviation in RA symptoms Reduction of CRP, and RF Decreased in inflammatory cytokines (TNF‐α and MCP‐1)	CIA mice model	[17]
13	hUCSCs and TNF‐α inhibitor	Reduced destruction of cartilage Increased in serum level of IL‐10	CIA mice model	[18]
14	combination of MSCs and bortezomib (a proteasome inhibitor)	A significant decrease in arthritis score	Animal model of RA	[19]
15	BM‐MSCs	Remission of the disease Regulation of inflammatory and anti‐inflammatory cytokines Reduced CRP level and ESR	CIA mice model	[20]
16	MSC‐derived EVs	Reduce proliferation of T and B cells Induction of Tregs differentiation Improvement in arthritis symptoms Prevention of plasma blast differentiation Induction of IL‐10‐expressing Breg	CIA mice model In vitro studies	[21]
17	BM‐MSC‐derived EVs	Inhibition of cyclin I/ATM/ATR/p53 signaling pathway by miR‐34a Suppression of aggressive FLS proliferation in joints	CIA mice model	[22]
18	MSC‐derived EVs	Suppression of aggressive FLS proliferation in joints Inhibition of CXCL9 chemokine Reduction of inflammation	CIA mice model	[23]
19	MSC‐derived EVs	Decreased Th17 cells Regulating the balance of Treg/Th17. Increase in TGF‐β expression	CIA mice model	[24]
20	hUC‐MSCs	Amelioration of the disease M2 polarization of macrophages Reduction of TNF‐α, and IL‐1	CIA mice model	[25]
21	MSCs	Amelioration of the disease Reduction of NF‐κB protein Increase of IκB protein in synovial fibroblasts production of TGF‐β1 Decrease in miR‐548e level	CIA mice model	[26]
22	BM‐MSCs and antioxidant (hesperidin) nd	Reduced free radical in the environment Improvement in survival and function of MSCs	AIA rat model	[27]

Abbreviations: AD‐MSC, adipose‐derived mesenchymal stem cells; AIA, adjuvant‐induced arthritis; BM‐MSCs, bone marrow mesenchymal stem cells; CIA, collagen‐induced arthritis; CRP, C reactive protein; CTLA4‐hAD‐MSCs, cytotoxic T‐lymphocyte antigen 4‐human adipose tissue‐derived mesenchymal stem cells; CTX‐II, C‐terminal cross‐linked telopeptide of type II collagen; CXCL9, C‐X‐C motif ligand 9; ESR, erythrocyte sedimentation rate; EV, extracellular vesicle; FLS, fibroblast‐like synoviocytes; Fox‐P3, forkhead box P3; GATA‐3, GATA binding protein 3; hUC‐MSCs, human umbilical cord‐mesenchymal stromal cells; hUCSC, human umbilical cord‐derived mesenchymal stromal cells; IL‐10, interleukin 10; IκB, inhibitor of NF‐κB; MCP‐1, monocyte chemotactic protein‐1; MIP‐2, macrophage inflammatory protein‐2; miR, microRNA; MSC, mesenchymal stem cells; NF‐κB, nuclear factor‐κB; RF, rheumatoid factor; SCID, severe combined immunodeficiency; T‐bet, T‐box expressed in T cells; TFH, follicular T helper; TGF‐β1, tumor growth factor‐β1; Th, T helper; TNF‐α, tumor necrosis factor‐α; Treg, regulatory T cell; UC‐MSC, umbilical cord mesenchymal stem cell.

**Table 2:** Results of stem cell therapy (SCT) in rheumatoid arthritis (RA) human patients.

Number	Type of stem cell	Result of SCT	Animal model/human	References
1	UC‐MSCs	IL‐10 secretion by UC‐MSCs Reducing expression of CDH11 on FLSs	CIA mice model In vitro studies	[28]
2	UC‐MSCs	Inhibiting proliferation, and invasiveness of FLSs by the production of IL‐10, and TGF‐β Reduction of MMP‐9 expression Inhibiting proliferation of T cells by production of PGE2, IDO‐1, and NO Induction of Tregs	RA patients	[14]
3	Autologous BM‐MSCs	Amelioration of the disease Reduced DAS28 index and ESR at 12 month after MSCT Decreased VAS score Significant decrease in RF level No significant change in CRP and anti‐CCP levels. Decrease of Th‐17 at 1 and 12 months Increase of Tregs after 1 month	Refractory RA patients	[29]
4	AD‐MSCs	Inhibiting production of inflammatory cytokines Inhibiting proliferation of collagen‐activated T cells Production of IL‐10 by MSCs MSCs‐induced production of IL‐10 by T cells and monocytes Induced differentiation of Tregs	RA patients	[9]
5	MSCs and MSCs‐differentiated chondrocytes	Suppressive effect on CII‐reactive T cells from RA patients Inhibition of T cell proliferation Inhibition of CD69 expression Suppression of TNF‐α, and IFN‐γ secretion from Th1, and CTL Increased secretion of IL‐4 and IL‐10	RA patients	[30]
6	Allogenic UC‐ MSCs	Inhibiting differentiation and proliferation of TFH in RA patients by production of IDO‐1 A balance between Treg/TH17 Induced production of TGF‐β	RA patients	[16, 24]
7	MSCs	Affecting memory T cells Induction of balance between inflammatory and anti‐inflammatory responses	RA patients	[31]
8	BM‐MSCs	Increase in mRNA expression of Fox‐P3 in PBMCs of patients Increase in the level of TGF‐β and IL‐10	Refractory RA patients	[32]
9	AD‐MSCs	Secretion of IL‐6, PGE‐2, and VEGF‐A Balance in Treg/Th17 ratio Increase in the production of TGF‐β Reduction in TNF‐α, IL‐21, and IL‐17 production	Co‐culture with PBMCs of RA patients	[33]
10	UC‐MSCs	Downregulation of ROR‐γ	Co‐culture with PBMCs of RA patients	[34]
11	AD‐MSCs	Inhibiting secretion of IL‐17 Inhibited expression of IL‐21 from PBMCs	Co‐culture with PBMCs of RA patients	[35]
12	epi‐hMSCs (MSCs with epigenetic change)	Production of lower level of IL‐17, and IFN‐γ	RA patients	[36]
13	UC‐MSCs and DMARDs	No adverse effects (safety of combination) Improvement in scores of ACR, DAS28, and HAQ‐DI (efficacy of combination) Elimination of joint pain, swelling, and stiffness after 12 months of treatment Decrease in CRP level and autoantibody titer Decrease in inflammatory cytokines such as IL‐6, and TNF‐α Increase in CD4 + CD25 + Foxp3+ regulatory T cells and Th2 producing IL‐4	Refractory RA patients	[37]
14	MSCs	No complications, including severe infections, malignancies or death Reduced DAS‐28, ESR, and VAS score 6 months after MSCT No complete remission and a disease relapse in long‐term	Refractory RA patients	[38]
15	hUC‐MSCs	No severe adverse effect in the short term Reduces DAS‐28 score, ESR, and inflammatory cytokines at 4 weeks after MSCT	RA patients	[39]
16	HSCs	Reduced disease activity based on DAS‐28 index in 50% of patients	RA patients	[40]
17	HSCs	Improvement in 67% of patients (ACR 50%) 6 months after HSCT Better response of RA patients with negative RF to HSCT treatment	RA patients	[41]
18	HSCs	Improvement of 39.4% of RA patients (ACR 70%)	RA patients	[42]
19	HSCs	Significant reduction in HAQ‐DI index in long‐term follow‐up 5‐year overall survival of 94% Disease‐free survival of 18%	RA patients	[43]
20	HSCs and high‐dose chemotherapy	Decrease of IgM RF and anti‐CCP titer after 3 and 6 months of HSCT Increase of IgM RF, and anti‐CCP titer after 1 year of HSCT Reduced immunoglobulin and CRP Reduced inflammation	RA patients	[44]
21	HSCs and high‐dose chemotherapy	Significant reduction in joint damage after 1 year of HSCT Decrease in CRP after 1 year of HSCT	RA patients	[45]

Abbreviations: ACR, American College of Rheumatology; AD‐MSCs, adipose tissue‐derived mesenchymal stem cells; AIA, adjuvant‐induced arthritis; BM‐MSCs, bone marrow mesenchymal stem cells; CCP, cyclic citrullinated peptide; CDH11, cadherin‐11; CIA, collagen‐induced arthritis; CRP, C reactive protein; CTL, cytotoxic T lymphocyte; DAS28, disease activity score 28; DMARDs, disease‐modifying antirheumatic drugs; epi‐hMSCs, MSCs with epigenetic change; ESR, erythrocyte sedimentation rate; FLS, fibroblast‐like synoviocytes; Fox‐P3, forkhead box P3; HAQ‐DI, Health Assessment Questionnaire‐Disability Index; HSCs, hematopoietic stem cells; HSCT, hematopoietic stem cell transplantation; hUC‐MSCs, human UC‐MSCs; IDO, indoleamine‐pyrrole 2,3‐dioxygenase; IFN‐γ, interferon‐γ; IgM, immunoglobulin M; IL‐10, interleukin 10; MMP‐9, matrix metallopeptidase 9; MSC‐derived EVs, MSC‐derived extracellular vesicles; MSCs, mesenchymal stem cells; MSCT, mesenchymal stem cell transplantation; NF‐κB, nuclear factor‐κB; NO, nitric oxide; PBMCs, peripheral blood mononuclear cells; PGE2, prostaglandin E2; ROR‐γ, RAR‐related orphan receptor‐γ; SCID, severe combined immunodeficiency; SCT, stem cell therapy; T‐bet, T‐box expressed in T cells; TFH, follicular T helper; TGF‐β, tumor growth factor‐β; Th, T helper; TNF‐α, tumor necrosis factor‐α; UC‐MSCs, umbilical core blood‐derived mesenchymal stem cells; VAS, visual analog scale; VEGF, vascular endothelial growth factor.

## 2 MSCT IN RA

### 2.1 MSCT in animal models of RA

Collagen‐induced arthritis (CIA) mice model is the most common animal model of RA disease.^46^ The RA disease is induced in mice by injection of complete Freund's adjuvant (CFA) and type II collagen (CII). Initial symptoms usually appear on Day 28 after injection.^47^ Injection of CII to mice causes irreversible erosions in bone, and cartilage.^48^

Intraperitoneal injection of allogeneic mesenchymal stem cells (MSCs) into the CIA mouse model prevented erosion of bone and cartilage. In addition, MSCT increased Tregs that contribute to the suppression of inflammation and amelioration of the disease severity.^4^, ^5^ Human umbilical cord‐MSCs (hUC‐MSCs) transplantation (UC‐MSCT) was used to evaluate its efficiency in CIA mice.^25^, ^49^ The results showed that UC‐MSCT improved the disease in CIA mice. Systemic administration of hUC‐MSCs ameliorated the disease in CIA mice due to M2 polarization of macrophages and reduction of TNF‐α and IL‐1β. Injection of UC‐MSCs to the mice reduced expression of inflammatory cytokines and chemokines such as TNF‐α, IL‐6, and monocyte chemoattractant protein‐1 (MCP‐1) and increased expression of anti‐inflammatory cytokines such as IL‐10.^49^ UC‐MSCs reduced Th1 and Th17 differentiation that have pathologic roles in RA. Moreover, Tregs were induced to suppress the immune response, Th1 and Th17 differentiation, and function. This leads to the decreased level of inflammatory cytokines, including interferon (IFN)‐γ and IL‐17.^6^, ^49^ Follicular T helpers (TFHs) that are a subtype of T cells contribute to the production of high‐affinity antibodies by affecting B cells. In RA, they have pathologic roles through the activation of autoreactive B cells and the production of autoantibodies.^50^ UC‐MSCT in CIA mice also reduced the frequency and stimulatory function of TFH. Moreover, in accordance with previous studies UC‐MSCs lead to the downregulation of inflammatory response.^7^, ^49^ UC‐MSC cells plus DMARDs therapy could be a safe, effective, and feasible therapeutic option for RA patients. One year and 3 years after UC‐MSC cell treatment, the blood routine, liver and kidney function, and immunoglobulin (Ig) examination showed no abnormalities. The erythrocyte sedimentation rate (ESR), C reactive protein (CRP), RF of 1 year and 3 years after treatment, and anti‐cyclic citrullinated peptide (CCP) of 3 years after treatment were detected to be lower than that of pretreatment, which showed significant change. Health index, health assessment questionnaire‐disability index (HAQ‐DI), and joint function index, disease activity score 28 (DAS28), decreased 1 year, and 3 years after treatment than before treatment.^51^

Jung et al. designed a study in which MSCT capability in CIA mice was evaluated. Results of liquid chromatography–tandem mass spectrometry‐based serum proteomics showed MSC improved inflammation through inhibition of platelet activation.^52^ In another study, Yan et al. showed MSCs interaperitoneally injected into CIA mice improved the disease in mice through the reduction of nuclear factor‐κβ (NF‐κB) protein and increased cytoplasmic inhibitor of NF‐κB (IκB) protein in the synovial fibroblasts by producing TGF‐β1. TGF‐β1 signaling also decreased microRNA (miR)‐548e level that binds to IκB protein and makes it inaccessible to synovial fibroblasts leading to suppression of NF‐κB activity.^26^ NF‐κB is a transcription factor playing a key role in inflammation and pathogenesis of RA and is responsible for the production of proinflammatory mediators in the synovium.^53^

Given the pathologic function of osteoclasts in the inflammatory milieu of synovium resulting in bone and cartilage destruction, the ameliorative effect of adipose‐derived mesenchymal stem cells (AD‐MSCs) on osteoclasts was evaluated in CIA mice. Injection of AD‐MSCs to mice prevented the destruction of the bone by inhibiting osteoclastogenesis and induction of immune tolerance in the mice.^8^ Accordingly, administration of human AD‐MSCs to CIA mice could reduce autoimmune responses followed by less damage to the cartilage accompanied by an improvement in arthritis.^8^, ^9^ These results suggest MSCs may show tolergenic properties resulting in the downregulation of autoreactive Th1 and Th17 responses. Moreover, they induce IL‐10‐secreting Tregs that demonstrate immunosuppressive roles.^54^ On the other hand, bone marrow MSC transplantation (BM‐MSCT) in CIA mice did not prevent the progression of the disease in mice and did not inhibit T cell proliferation.^10^ Interestingly, the results of an in vitro study were different showing BM‐MSCs had an inhibitory effect on activated T cells by IFN‐γ‐dependent pathway leading to the induction of nitric oxide (NO), and prostaglandin E2 (PGE2) preventing T cell proliferation.^10^, ^55^, ^56^ The controversy could be explained through different milieu of in vivo and in vitro. Indeed, in vivo contains a more complex microenvironment, while the conditions in vitro are more controlled compared to in vivo ones. In another study, the effect of UC‐MSCs on the expression level of cadherin‐11 (CDH11) was examined in a transwell system and CIA mice.^28^ CDH11 is an adhesion molecule that induces FLSs to form a lining layer and promote the invasive behavior of FLSs. FLSs express high levels of CDH11 resulting in inflammation and joint damage.^57^, ^58^ The results showed that IL‐10 secretion by UC‐MSCs reduces the expression of CDH11 on FLSs in the culture system and in the synovium of CIA mice.^28^ Therefore, MSCs could alter the expression of FLSs adhesion molecules in the joints and contribute to the amelioration of RA disease.

In some studies, the therapeutic efficacy of engineered MSCs was evaluated in RA treatment. For example, the therapeutic effect of genetically modified MSCs overexpressing IL‐10 was evaluated in mouse CIA. The results showed that IL‐10‐MSCs inhibited the initiation of the disease and reduced the severity of the arthritis. Inhibition of T cell proliferation in response to CII as well as a decrease in IL‐6 and anti‐CII antibodies were observed.^11^ In another study, engineered MSC progenitors expressing Brachyury which is a member of the T‐box transcription factor were evaluated for their effects on the cartilage tissue in the presence of the aggressive fibroblasts in rheumatoid synovium of severe combined immunodeficiency (SCID) mice. The SCID mice are severely deficient in T and B cells. The engineered MSCs and aggressive rheumatoid arthritis synovial fibroblasts (RASFs) were injected into the mice. RASFs have a major role in RA by contributing to pannus formation and containing an aggressive phenotype invading the healthy cartilage. The results showed the formation of new cartilage tissue. Indeed, the aggressive and destructive RASFs could not prevent the cartilage formation, and destroy the cartilage in the presence of engineered Brachyury‐MSCs.^12^, ^13^ Another engineered MSC containing TGF‐β (TGF‐β‐MSCs) was evaluated for the treatment of CIA mice. The results showed an amelioration in arthritis accompanied by inhibition of osteoclast differentiation. Moreover, a balance in Tregs/Th17 ratio and reduction in proinflammatory cytokines were detected.^14^

Cytotoxic T‐lymphocyte antigen 4 (CTLA‐4)‐Ig has an immunosuppressive effect and can inhibit the pathogenic process of RA. In a study, a human amnion‐derived mesenchymal stem cell (hAD‐MSC) was engineered to express CTLA‐4. Thereafter, the therapeutic effect of xenogeneic CTLA‐4‐hAD‐MSCs was evaluated in CIA mice and compared with syngeneic AD‐MSCs. The results showed a decrease in severity of the cartilage injury in mice injected with xenogeneic CTLA‐4‐hAD‐MSCs as well as a reduction in the serum level of C‐terminal cross‐linked telopeptides of type II collagen (CTX‐II). CTX‐II is considered a potential biomarker for cartilage degradation and disease progression in RA. On the other hand, these effects were not detected in the group receiving syngeneic hAD‐MSCs.^15^, ^16^ These results suggest that engineering of hAD‐MSCs with CTLA‐4, though xenogeneic, may be more effective in the treatment of RA patients than syngeneic AD‐MSCs alone. The effect of treatment with CTLA4‐Ig‐hAD‐MSCs was also compared with hAD‐MSCs alone in CIA mice. The results confirmed a decrease in arthritis score and serum level of IL‐12, and macrophage inflammatory protein 2 in the knee‐joint extract of CTLA‐4‐Ig‐hAD‐MSCs mice than hAD‐MSCs mice. In vitro studies showed both hAD‐MSCs and CTLA‐4‐Ig‐hAD‐MSCs treatment significantly decreased the expression of T‐box expressed in T cells (T‐bet) and GATA binding protein 3 (GATA‐3) transcription factors in splenocytes from arthritic mice. Moreover, CTLA4Ig‐hAD‐MSCs treatment significantly increased the ratio of CD4 + CD25 + FoxP3 + Treg/CD4 + CD25 + RORγt + Th17 cells.^16^ These studies suggest engineered CTLA‐4‐Ig‐hAD‐MSCs could be an effective therapeutic option in RA patients.

In a study, a combination containing MSCs and IL‐4 was more effective than MSC therapy alone in CIA mice. Mice that received the combination showed an alleviation in RA symptoms and a reduction in the levels of CRP and RF. Inflammatory cytokines, including TNF‐α and MCP‐1 were decreased.^17^ Interestingly, Wu et al. showed that articular injection of human umbilical cord‐derived mesenchymal stromal cells (hUCSCs) alone did not have any significant effect on the disease improvement in CIA mice. However, injection of hUCSCs with TNF‐αi could reduce the destruction of cartilage and increase serum IL‐10 suggesting TNF‐α inhibits the therapeutic effect of hUCSCs. Therefore, a combination containing hUCSCs and TNF‐αi could be useful in the treatment of RA.^18^ Accordingly, Papadopoulou et al. found out inflammatory milieu of joints in RA could suppress anti‐inflammatory effects of MSCs. Herein, a combination containing MSCs and bortezomib (a proteasome inhibitor) was used in the animal model of RA. The results showed a significant decrease in arthritis score. On the other hand, infusion of MSCs or bortezomib alone did not show significant effects.^19^ The results of another study showed that IFN‐γ is a key factor in determining the efficacy of MSCT in the treatment of RA. MSC plus IFN‐γ combination therapeutic strategy can greatly improve the clinical efficacy of MSC‐based therapy in RA patients.^59^ Altogether, the addition of some cytokines such as IL‐4 or cytokine inhibitors such as TNF‐αi can increase the efficacy of MSCs therapy for RA patients; however, more investigations are required to address this issue.

The therapeutic efficacy of MSCs (BM‐MSCs) has been compared with conventional immunosuppressive drugs (betamethasone) in CIA mice. Interestingly, BM‐MSCs showed more remission impact than betamethasone in CIA mice. Evaluation of BM‐MSCT on immunological markers showed BM‐MSCs regulated expression of inflammatory and anti‐inflammatory cytokines and significantly reduced CRP level and ESR.^20^ MTX therapy in some RA patients does not show an impact on improvement of the disease. The efficacy of MSCT, MTX, and both of them, was evaluated in CIA mice. Combined usage of MSCs and MTX increased the therapeutic efficacy of RA, decreased arthritis score, inflammatory responses, and mortality.^60^

MSCs not only release anti‐inflammatory mediators but also release extracellular vesicles (EVs) that are transported into the blood. EVs contain a phospholipid bilayer, a large variety of proteins, messenger ribonucleic acids (mRNAs), and miR. In vitro and in vivo evaluation of possible immunosuppressive effects of two main types of EVs, including exosomes and microparticles on T and B cells in CIA mice showed both exosomes and microparticles reduce proliferation of T and B cells and induce differentiation of Tregs. However, exosomes had more therapeutic effect on arthritis symptoms in CIA mice than microparticles. The higher therapeutic efficacy of exosomes may be due to the prevention of plasma blast differentiation, and induction of IL‐10‐expressing Breg cells in lymph nodes.^21^ Moreover, BM‐MSC‐derived EVs contain miR‐34a that affects the cyclin I/ATM/ATR/p53 signaling pathway resulting in the suppression of aggressive FLS proliferation in joints and amelioration of RA.^22^ Accordingly, another miR, miR‐320a, in exosomes contributes to the suppression of FLS proliferation by inhibiting C‐X‐C motif ligand 9 chemokine resulting in the reduction of inflammation.^23^ Comparison of MSC treatment alone with MSC‐derived EVs treatment showed EVs treatment is safer and more effective in alleviating inflammation in CIA mice.^61^ On the other hand, Ma et al. compared the immunomodulatory effects of EVs with MSCs. The results showed no significant difference between the two groups. Both groups decreased Th17 cells and regulated the balance of Treg/Th17. Moreover, despite an increase in TGF‐β expression by both groups, MSCs were more effective in increasing TGF‐β than EVs.^24^ In a genetically engineered study, the effect of MSCs‐derived exosomes containing miR‐146a/miR‐155 on the lymphocyte population and function was evaluated in CIA mice. Reduced IFN‐γ expression in CIA mice after treatment, suggested that manipulation of MSCs‐derived exosomes could decrease proinflammatory cytokine production to strike a balance among Th subtypes.^62^ In another study, exosomal miR‐205‐5p from MSC introduced a therapeutic RA target. MiR‐205‐5p expressed in chondrogenic BM‐MSCs‐derived exosomes highly inhibited, joint destruction and inflammation, and suppressed mitogen‐activated protein kinases and NF‐κB pathways through murine double minute 2 (a nuclear‐localized E3 ubiquitin ligase) in CIA mice.^63^ Another SCT confirmed modulatory roles for exosomes derived from MSC in RA by creating a balance between TH17, and Treg differentiation. Injection of these exosomes into CIA mice was associated with reduced swelling in paws, hyperplasia in synovial, decreased inflammatory cytokines, and anti‐collagen IgG levels in serum. Furthermore, elevated TGF‐β, IL‐10, and reduced IL‐17 contribute to restoring the balance between TH17, and T reg.^64^ Long noncoding RNAs (lncRNAs) are pathogenic in several organs such as heart and neural crest derivatives, expressing 2‐antisense RNA 1 (HAND2‐AS1) in RA‐FLSs and is associated with progression of RA. Functions of HAND2‐AS1 as an exosomal lncRNA related to MSCs evaluated in RA treatment. Results showed overexpression of HAND2‐AS1 in exosomes derived from MSCs, induce inactivation of NF‐κB pathway via miR‐143‐3p/TNFAIP3 axis. Proliferation, invasion, migration, inflammation, and apoptosis are suppressed in RA‐FLSs.^65^ Gingival mesenchymal stem cells (G‐MSCs) have immunotherapy potential, however, accompanied with immunogenicity and tumorigenicity. Tian et al. investigated immunosuppressive effect of G‐MSC with G‐MSC‐derived exosomes (G‐MSC‐Exo). Results show that injection of G‐MSC/G‐MSC‐Exo leads to mitigating inflammation and erosion of bone in CIA mice and G‐MSC‐Exo has identical or stronger capability for effects. G‐MSC‐Exo plays a role by regulating the imbalance between Th17/Treg in vitro or in vivo and suppressing IL‐17RA‐Act1—tumor necrosis factor receptor‐associated factor 6—NF‐κB signaling pathway in vivo.^66^ Huang et al., showed miR‐223 encapsulated in exosomes and secreted by B‐MSCs, inhibit immune response against the progression of RA. Effects of exosomes included inhibiting the release of IL‐1β, TNF‐α, and IL‐18. MiR‐223 can suppress the NLR family pyrin domain containing 3 activations in macrophages and RA rats.^67^ Therapeutic effects of EVs derived from MSC were examined in an adjuvant‐induced arthritis (AIA) model. EVs isolated from MSCs were cultured normoxically (21% O_2_, 5% CO_2_), and the AIA model was applied with normoxic EVs. These are associated with the establishment of TH17/Treg balance that resulted in reduced inflammation.^68^

Considering the pathological situation of RA, survival, proliferation, and function of stem cells were impaired. Zhao et al. suggested an infliximab‐based self‐healing hydrogel composite scaffold as a new therapeutics. The composite scaffolds, along with ADSCs, are then implanted into the critical‐sized bone defect in the RA rabbit model. Downregulation of inflammatory cytokines, rebuilding damaged cartilage, as well as improved subchondral bone repair, confirm the ability of bioengineered composite scaffolds.^69^

### 2.2 Effect of MSC origin on the therapeutic efficacy of RA and limitations of MSCs

Immunosuppressive functions of BM‐MSCs, UC‐MSCs, and AD‐MSCs were compared in a study, and the optimal dose for induction of more Tregs was determined in CIA mice. The results showed that BM‐MSCs had a greater therapeutic effect and induced more Tregs in mice than the other two types of MSCs. The optimal dose of MSCs for induction of Tregs in CIA mice was reported to be 5 × 10^6^ cells.^70^ Therefore, the origin of MSCs could affect the therapeutic efficacy of RA. In addition, the therapeutic effects of MSCs, HSCs, and MTX were compared in another study in the RA rat model. The results showed that MSCs are more effective for the improvement of RA symptoms, and RA scores resulting in a decrease in RF levels and inflammatory cytokines.^71^ MSC therapies have been used as cell‐based treatments for decades, due to their anti‐inflammatory, immunomodulatory, and regenerative properties. The largely positive outcomes in clinical trials without severe side effects establish MSCs as promising tools for arthritis treatment.^72^

Short survival and low immunomodulatory effects of MSCs in the oxidative stress environment are one of the MSCs limitations.^73^ In a study, BM‐MSCs were injected with an antioxidant (hesperidin) into AIA rats, another animal model of RA. Hesperidin reduced free radicals in the environment and improved the survival and function of MSCs.^27^ Therefore, injection of an antioxidant in MSC therapy may help to increase the survival of MSCs.

### 2.3 MSCT in RA patients

In an interesting study, the characteristics of BM‐MSCs in people with RA and healthy people were compared. Results indicated molecular, proteomic, morphologic characteristics, immunophenotype, and frequency of MSCs in BM of RA patients are not different from healthy people. Therefore, the therapeutic effect of autologous MSCT could be similar to that of allogenic MSCT in the improvement of RA patients; but autologous MSCs of RA patients showed reduced proliferative potential that is related to a decrease in the telomere length and changes in the expression of genes involved in focal adhesion and cell cycle pathways.^74^

Investigating the effect of UC‐MSCT on T cells and FLSs from RA patients showed UC‐MSCs inhibited proliferation, and invasiveness of FLSs by the production of IL‐10, TGF‐β, and reduction of matrix metalloproteinases 9 expression. In addition, they suppressed the proliferation of T cells by production of PGE2, indolamine‐2,3‐dioxygenase 1 (IDO‐1), and NO and induced Tregs in RA patients.^6^

Autologous BM‐MSCT resulted in the amelioration of the disease in refractory RA patients. A single infusion of BM‐MSCs reduced the DAS28 index which is a measure of disease activity in RA. Moreover, a reduction in ESR after 12 months of MSCT and a decrease in the visual analog scale (VAS) score that usually used to measure the pain score, was detected. Flow‐cytometry results showed Th‐17 decreased after BM‐MSCT and Tregs increased. There was a significant decrease in serum RF after MSCT; however, a significant change was not detected in CRP and anti‐CCP levels.^29^ Another human study was designed to determine immunosuppressive features of collagen‐reactive T cells obtained from RA patients. AD‐MSCs inhibited the production of inflammatory cytokines as well as reduced the proliferation of collagen‐activated T cells. Accordingly, AD‐MSC not only produced IL‐10, but also motivated T cells and monocytes for the production of IL‐10, and differentiation of Tregs.^9^ Accordingly, MSCs and MSCs‐differentiated chondrocytes have suppressive effects on CII‐reactive T cells from RA patients. The suppressive effect of these cells could be mediated in different ways, including inhibition of T cell proliferation, expression of CD69, suppression of TNF‐α, and IFN‐γ secretion from Th1 and CTL, and increased secretion of IL‐4 and IL‐10.^30^

AD‐MSC from RA patients synovial are capable of differentiation to adipocytes, chondrocytes, and osteoblasts. AD‐MSC cells also can inhibit CD4+ T cell proliferation.^75^

TFH cells increase in the peripheral blood (PB) of RA patients and its number is associated with anti‐CCP autoantibody level. Allogenic UC‐MSCs inhibited differentiation and proliferation of TFH in RA patients by production of IDO‐1. They created a balance between Treg/TH17 and induced production of TGF‐β. Given the role of TFH and TH17 in the pathogenesis of RA, suppression of these cells could be helpful in the alleviation of RA disease.^7^, ^24^ MSCs could affect memory T cells that in turn could modify the balance between inflammatory and anti‐inflammatory responses in RA patients.^31^ Evaluation of refractory RA patients after 12 months of the BM‐MSCT showed an increase in mRNA expression of forkhead box P3 (FOX‐P3) (a crucial transcription factor regulating Tregs gene expression) in peripheral blood mononuclear cells (PBMCs) of patients suggesting an increase in Treg differentiation and subsequent increase in the level of TGF‐β and IL‐10. These alterations may be associated with an improvement in the disease course and activity.^32^ Co‐culture of AD‐MSC with PBMCs of RA patients induced secretion of soluble factors such as IL‐6, PGE‐2, and vascular endothelial growth factor A. In addition, a balance between Tregs and Th17 cells was established in which a skewness towards the differentiation of Tregs occurred. In addition, an increase in the production of TGF‐β and reduction in proinflammatory cytokines, including TNF‐α, IL‐21, and IL‐17 was detected. These effects may prevent the progression of the disease.^33^ Accordingly, co‐culture of UC‐MSCs with PBMCs from RA patients downregulated mRNA expression and protein levels of RAR‐related orphan receptor gamma (a crucial regulator of Th17 gene expression), especially in severe RA patients suggesting a suppressive effect of UC‐MSCs on Th17 differentiation.^34^ Consistently, co‐culture of AD‐MSCs with PBMCs from RA patients inhibited secretion of IL‐17. In addition, it prevented the expression of IL‐21 from PBMCs owing to the regulatory function of AD‐MSCs.^35^ Altogether, these results suggest MSCs as a candidate for cell‐based therapy in RA patients.

Epigenetic changes in human MSCs could affect its immunoregulatory properties and expression of molecules, including IL‐10, and IDO‐1 that enhance immune suppression. These epigenetic changes in MSCs have inhibitory effects on Th‐17 cells differentiation, T cell proliferation, and cytokine production. Mononuclear cells from synovial fluid of RA patients that were treated with these MSCs produced lower levels of IL‐17 and IFN‐γ. These results suggest epigenetic changes in MSCs could alter its therapeutic effect in RA patients.^36^

**Figure 2:**
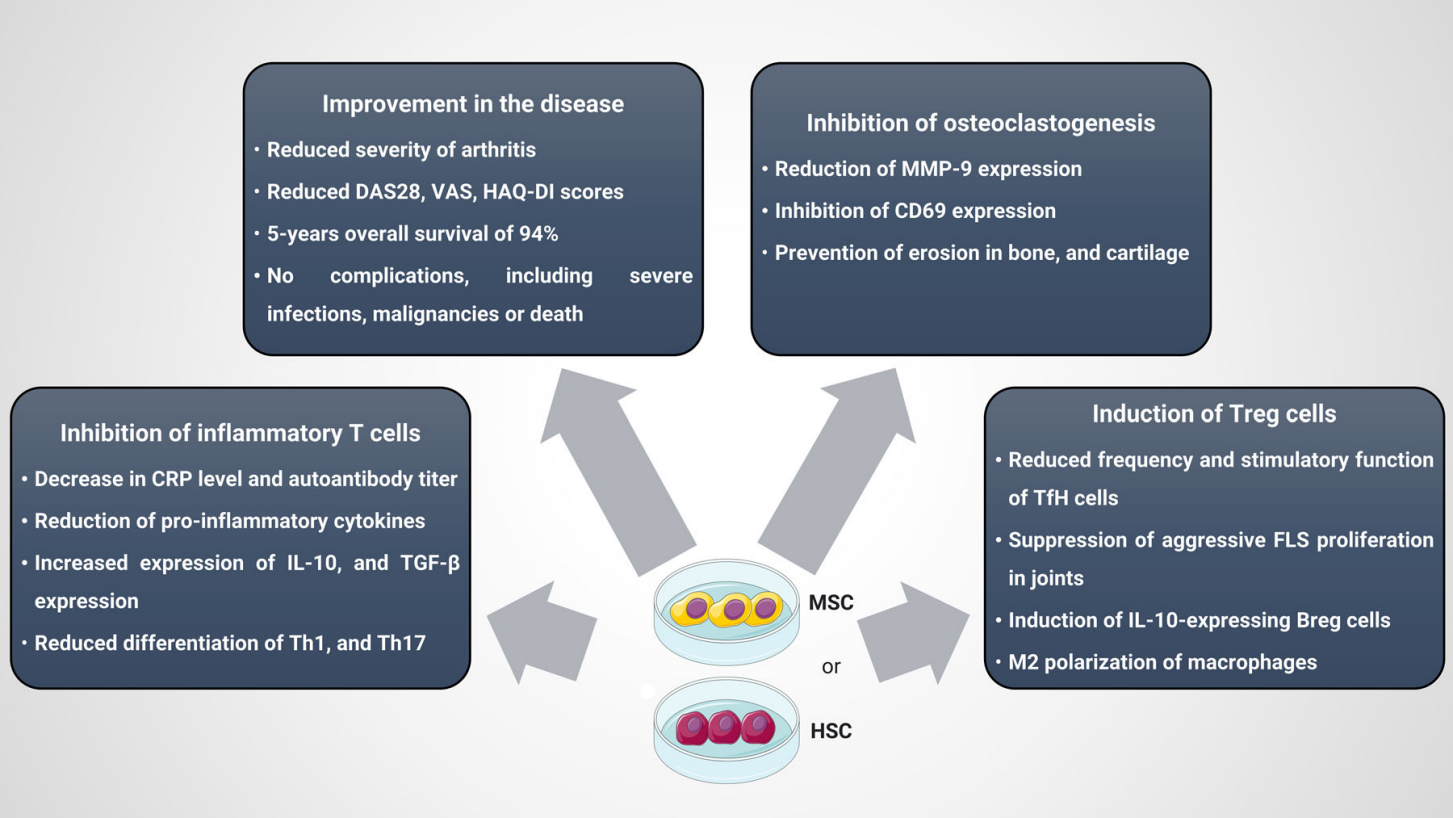
Therapeutic effect of MSC, and HSC in RA animal models/human patients. The protective effects of MSCT/HSCT have been summarized in the figure. These effects include inhibition of inflammation, Treg induction, improved clinical aspect of the disease, and inhibition of osteoclastogenesis. DAS28, disease activity score 28; FLS, fibroblast‐like synoviocytes; HAQ‐DI, Health Assessment Questionnaire‐Disability Index; HSC, hematopoietic stem cells, HSCT, hematopoietic stem cell transplantation; IL‐10, interleukin 10; MMP‐9, matrix metallopeptidase 9; MSC, mesenchymal stem cell; MSCT, mesenchymal stem cell transplantation; RA, rheumatoid arthritis; TFH, follicular T helpers; TGF‐β, tumor growth factor‐β; Th, T helper; Treg, regulatory T cell; VAS, visual analog scale.

The therapeutic efficacy of a combination containing MSCs with the other drugs was evaluated in some studies. A combination consisting of UC‐MSCs and DMARDs (conventional therapy) in refractory RA patients showed that the treatment is a safe and effective method for a long time in refractory patients. No adverse effects were observed and scores of American College of Rheumatology (ACR), a scale to measure change in RA symptoms, DAS28, and HAQ‐DI, the gold standard method for the assessment of function in patients with RA, the clinical variable most closely associated with joint replacement, work disability, and mortality improved. Joint pain, swelling, and stiffness were eliminated after 12 months of treatment. In addition, CRP level, autoantibody titer, and serum inflammatory cytokines such as IL‐6, and TNF‐α declined. On the other hand, CD4 + CD25 + FoxP3+ Treg and Th2 producing IL‐4 increased in PB of the patients. DMARDs alone were not enough effective in the improvement of the disease in the patients.^37^ Ahmed. et al. evaluated effect of MSC, curcumin, and their combination on serum cytokines and gene expression in CFA‐induced arthritis in male and female Wistar rats. After therapy, serum levels of IL‐17, PGE2, and IL‐13 returned to the normal level, and gene expression of IL‐6, and COX‐1 increased and IL‐13 decreased. These change could be effective on the reduction of inflammation; and this reduction with a combination of them is more effective than their individual use, also having advantageous effects on the ankle joint, thymus, and spleen (Figure 2).^76^ Kevin Sheng‐Kai Ma demonstrated a combination of human umbilical cord mesenchymal (stromal) stem cell (hUC‐MSCs) transplantation with IFN‐γ treatment synergistically improves the clinical outcomes of patients with RA.^59^ Small interfering RNAs (siRNA) can inhibit the expression of specific genes. Panet et al. examined the effects of simultaneous therapy with both BM‐MSC and siRNAs targeting IL‐1β/TNF‐α in CIA model rats. They concluded the therapy with siRNA and BM‐MSC showed synergism effects on alleviating the inflammation and cartilage repair of RA rats.^77^

### 2.4 Safety of MSCT in RA patients and prediction of MSCT outcome

Monitoring of refractory RA patients transplanted with MSCs performed by Liang et al. showed no complications in the patients, including severe infections, malignancies, or death suggesting MSCT could be considered as a safe therapeutic module for RA patients. DAS‐28, ESR, and VAS scores were reduced in 6 months after MSCT. However, long‐term follow‐up showed none of the patients achieved complete remission and experienced disease relapse. The unsuccessful outcome of MSCT in long‐term may be due to the low number of infused MSCs in the patients.^38^ Evaluation of short‐term MSCs safety in RA patients in the 2018 phase I trial showed a single intravenous infusion of hUC‐MSCs to RA patients do not have any serious adverse effects in the patients. Moreover, it reduces DAS‐28 score, ESR, and inflammatory cytokines at 4 weeks after MSCT.^39^ Wang et al. evaluated efficacy of a combination consisting of UC‐MSC and DMARDs in refractory RA patients. They found out the treatment was safe in long‐term, and no adverse effects were observed.^37^ Altogether, these studies suggested the safety of MSCT in RA patients; however, more studies are necessary to address the issue.

With accumulating evidence and evolving technology, cell‐based therapy has shown initial therapeutic potential for RA. However, the implementation of cell‐based therapy in clinical practice requires extensive preclinical and clinical studies, because such a new practical treatment strategy still faces many challenges. Most studies of MSC‐based therapies focus on evaluating safety and efficacy, while MSC composition, route of administration, dose, and frequency remain undefined. In addition, it is unknown whether MSC transplantation is effective only for a certain number of disease phenotypes, as MSC transplantation has not shown a significant benefit in refractory RA. Furthermore, although MSCs have been reported to induce significant T‐cell immunosuppression in severe inflammation, a less inflammatory environment can alleviate T‐cell suppression by MSCs and even promote T‐cell responses.

To predict the outcome of allogenic MSCT, the level of IFN‐γ was evaluated. The results showed as high as IFN‐γ level, the better outcome and success of MSCT. This is probably due to the induction of IDO‐1 by IFN‐γ.^78^ Indeed, the higher IFN‐γ is accompanied by more safety, efficacy (based on DAS‐28, and ACR indexes), reduction of inflammatory mediators, and recovery of RA patients from the disease.^79^ Therefore, the level of IFN‐γ may be used to predict the MSCT outcome.

## 3 HSCT IN RA

Bekkum et al. showed bone marrow transplantation (BMT) leads to a clear curative effect in the progressive stage of RA in rats. In this study, BMT was accompanied by total body irradiation as a conditioning regimen resulting in a long‐term remission of the disease. This study introduced HSCT as a potential therapeutic option for RA. Early human studies in this area were conducted on a limited number of human patients reporting the results as case report studies. Jacobs et al. in 1986 and Joske et al. in 1997 performed the first HSCT in human patients with RA, including one RA woman with aplastic anemia and another one with RA man, respectively. The results of both studies demonstrated an improvement in RA symptoms. These studies paved the way for more studies on the therapeutic effects of HSCT in RA.^80^, ^81^, ^82^

### 3.1 HSCT in RA patients

In HSCT, cyclophosphamide is often used as a conditioning regimen before transplantation. Efficacy and response of RA patients to the treatment with HSCT are evaluated by measurement of molecular markers, and evaluation of clinical indexes, including DAS‐28, ACR, and HAQ‐DI. Based on the DAS 28 score, Verburg et al. reported that 50% of RA patients showed significantly reduced disease activity.^40^ Snowden et al. demonstrated HSCT resulted in ACR 50%, after HSCT (6 months) in 67% of patients with RA. The results also showed patients with negative RF respond better to HSCT treatment.^41^ Therefore, RF level could be considered as a predictor for treatment outcome. In another study, 39.4% of RA patients acquired ACR 70% after HSCT.^42^ Another criterion used to assess the functional status of patients with RA is the HAQ‐DI. Long‐term follow‐up of RA patients treated with HSCT showed a significant reduction in the HAQ‐DI index.^41^, ^83^ Moreover, 5‐year overall survival and disease‐free survival of the patients was 94% and 18%, respectively, after HSCT.^43^ Altogether, these results suggest HSCT could partly improve the disease in some RA patients.

Verburg et al. examined the immunological effect of HSCT as well as high‐dose chemotherapy (HDC) in synovial tissue and PB of RA patients. IgM RF and anti‐CCP titer decreased at 3, and 6 months after transplantation, but they increased again after 1 year. This is probably due to incomplete removal of autoreactive B cells from the body that produce autoantibodies and leads to failure of long‐term recovery of RA patients after transplantation. On the other hand, human Ig, CRP, and inflammation were reduced in responder patients.^44^ In another study, the effect of HSCT and HDC on the joint damage was examined. Herein, a significant reduction in the joint damage and decrease in CRP was observed in the first year of transplantation suggesting a reduction in the infiltration of T cells into the synovium. The consequent is inhibition of osteoclast activation and damage to the joints.^45^

Considering that the reduction of autoreactive T cells in the infused HSCs may increase the efficacy of the treatment, two groups of patients were transplanted with differential HSCs: one group transplanted with CD34+ stem cells depleted from T cells and another group received unmanipulated HSCs. The results showed the ACR score and the duration of remission are not significantly different between the two groups suggesting T cell depletion from HSCs does not have a significant effect on the efficacy of HSCT treatment (Figure 2).^84^

### 3.2 Limitation of HSCT in RA patients and prediction of HSCT outcome

Several studies showed the rate of toxicity and death in severe RA patients receiving a high dose chemotherapy followed by HSCT were low suggesting the safety of this treatment.^40^, ^41^, ^83^ Despite the safety and partial efficacy of HSCT and HDC, the early recurrence of the disease is common. Moore et al. showed that RF‐positive patients are more likely to have a recurrence of the disease after HSCT. During the recurrence of the disease, a strong immune suppressive conditioning regimen is used to kill autoimmune memory cells and consequently control the disease despite its risk of increasing infections and death.^42^

Immunohistochemistry results of synovium show patients that express high levels of CD3, CD4, CD27, and CD45 on their T cells in synovium tissues, respond better to the treatment, 3 months after HSCT. After HSCT, a decrease in T cells occurs accompanied by a partial improvement in the disease. On the other hand, patients who lacked T cells in their synovium tissues and produced a high amount of IL‐1 are nonresponder to HSCT treatment.^44^ These results suggest immunological profile of synovium tissues obtained by biopsy could be a predictor of outcome in HSCT treatment.

## 4 EMBRYONIC STEM CELLS (ESCs) IN RA

The inner cell mass of the human blastocyst, an early stage of the developing embryo lasting from the fourth to the seventh day after fertilization, contains ESCs. They vanish after the seventh day and start to form the three layers of embryonic tissue in a typical development. For the first time in 1998, human ESCs were successfully expanded in the lab.^85^ Beneath suitable culture conditions, ESCs have illustrated an exceptional capacity to self‐renew persistently, that is, to create more cells like themselves that are multipotent.

Transplantation studies show that ESCs have immunosuppressive effects and can inhibit local immune response through cell‐to‐cell contact.^86^ Due to the finiteness of adult MSCs and the long‐term protocol of their expansion, and genomic instability occurs and antigenicity of MSCs increases. In a study, the effect of MSCs derived from human embryonic stem cells (hESC‐MSC) was to be investigated in the recovery of mice with CIA. The results showed that treatment with hESC‐MSC induces Treg cells, Th1, and IDO‐1 which reduces the severity, and progression of the disease.^87^

Other cells whose therapeutic effect has been studied in RA are cells positive for stage‐specific embryonic antigen‐3, which acts as stem cells in the blood. In this study, Kurose et al. found that these cells exist in the synovial tissue even in pathological conditions such as RA. After isolating these cells from the synovial of RA patients, they were cultured and transferred intravascularly to AIA mice. They witnessed their inhibitory effect on arthritis and joint destruction.^88^

## 5 HUMAN AMNIOTIC MEMBRANE CELLS (HAMCs) IN RA

Cells isolated from HAMCs display immunosuppressive roles in vitro such as impairing lymphocyte proliferation, activation, dendritic cell (DC) maturation, inflammatory cytokine production, induction of Treg, and M2 macrophage. Immune suppression properties of these cells were investigated through the treatment of cells isolated from RA patients with HAMCs in CIA mice treated with HAMCs. Results showed an inflammatory synovial response and TH1/TH17 pathway suppressed in isolated PBMC from RA patients. In treated CIA mice, the production of inflammatory chemokines, and cytokines was reduced, and the generation of peripheral Treg was induced. Therefore, HAMCs may be an attractive candidate in the cell‐based therapy for RA.^89^, ^90^

## 6 Treg CELLS IN RA

### 6.1 Treg cells and their effects

Treg cells were classified into three categories based on their origin and differentiation: Natural Tregs (nTregs), are generated from developing T lymphocytes in the thymus with phenotype of CD4 + CD25 + Foxp3+ T cells. They constantly express CD25 and Foxp3 which is a transcription factor. After induction of immune suppressive factor, mature CD4 + CD25− T cells are converted into inducible Treg cells, including Tr1 and Th3 subsets. Tr1 mainly produces IL‐10, and TGF‐β, and Th3 generates TGF‐β. Besides regulatory CD4+ T cells, regulatory CD8+ T cells also exist.^91^, ^92^

The inhibitory effect of Treg cells mediated through T cell immunoreceptor with Ig and immunoreceptor tyrosine‐based inhibitory motif domains on the surface of Treg cells and its interaction with CD155 on DCs surface. This is followed by an increase in IL‐10 expression and a decrease in IL‐12 production in DCs, resulting in the prevention of effector T‐cell activation.^93^, ^94^

T‐bet which is a transcription factor associated with Th1 cells, is related to the TIGIT expression. The T‐bet + TIGIT+ Treg phenotype prevents the proinflammatory immune response mediated by Th1 and Th17 cells.^95^, ^96^ Th2‐related transcription factor, IRF‐4, induces the expression of co‐stimulatory molecules, including CTLA‐4 and ICOS, in Treg cells and cooperates with recombination signal binding protein for Ig kappa J region and JUNB to prevent the immune response related to Th2 cells.^97^, ^98^, ^99^

Treg cells express some surface markers, such as glucocorticoid‐induced TNFR‐related protein (GITR), CTLA‐4, programmed death‐1 (PD‐1), and its ligand (PD‐L1).^100^ CTLA‐4 binds to CD80, and CD86 on antigen‐presenting cells (APCs) (especially DCs). These bindings prevent the antigen presentation and maturation of APCs and increase the expression of IDO in DCs, decreasing the tryptophan necessary for effector T cell proliferation.^101^, ^102^ PD‐1 binds to PD‐L1 and PD‐L2 ligands on DCs to inhibit effector T cells.^103^ Lymphocyte activation gene 3 binds to major histocompatibility complex (MHC)‐II, negatively regulating the function of T cells, and preferentially inhibits the response of T cells to the MHC‐II.^104^, ^105^ The transmembrane protein glycoprotein‐A repetitions predominant (GARP)/latency‐related peptide is related to the capability of Treg cells to activate TGF‐β after stimulation by T cell receptor (TCR).^106^ Downregulation of GARP expression reduces the inhibitory function of Treg cells.^107^ TNF‐related apoptosis‐inducing ligand (TRAIL) is expressed when Treg cells are activated, while CD4+ effector cells express its ligand death receptor 5 (DR5). The TRAIL/DR5 binding activates caspase‐8 to induce the apoptosis of effector lymphocytes.^108^, ^109^

**Figure 3:**
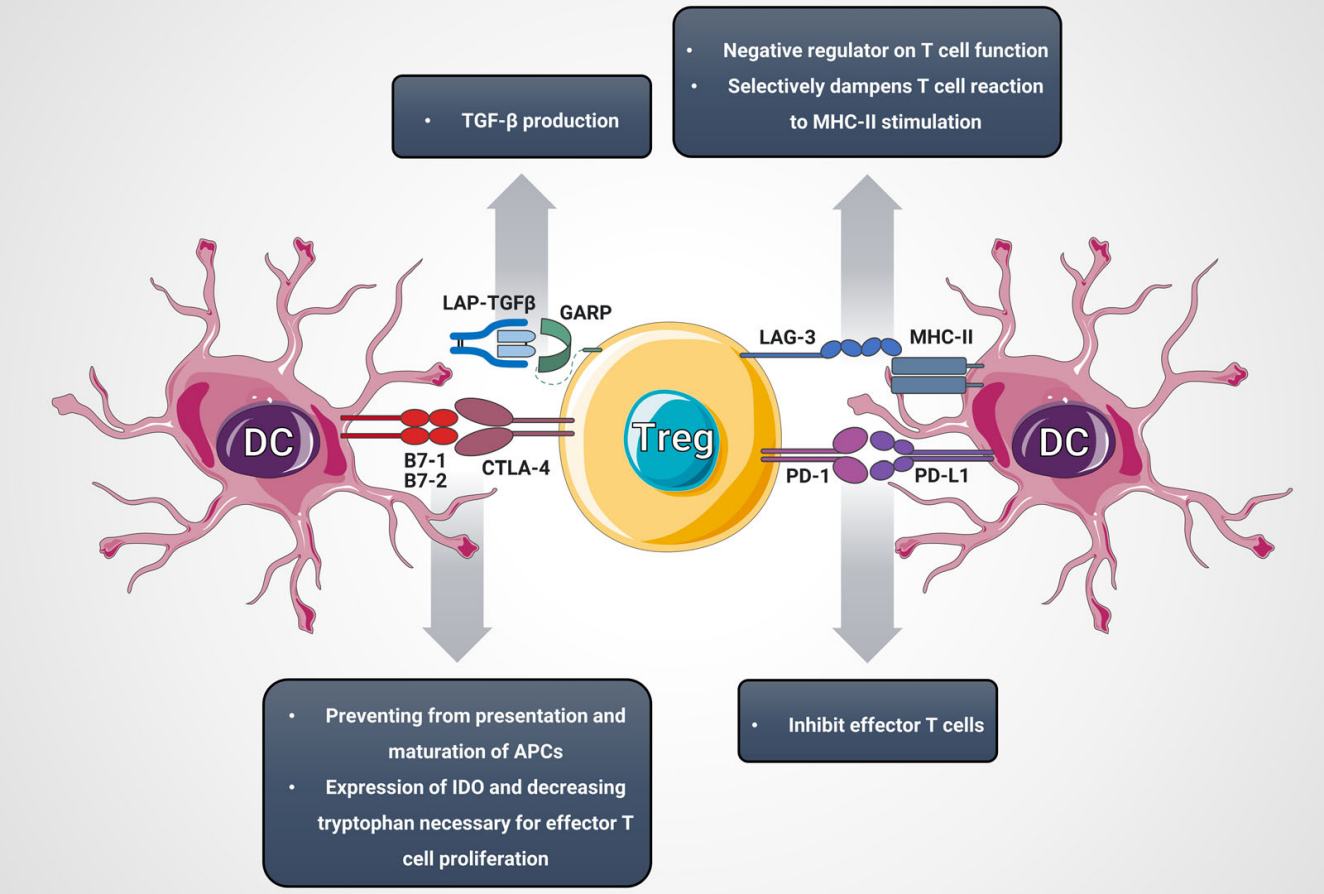
Molecular markers of Treg and their interactions with DCs molecular markers. Inhibitory ligands/receptors of Treg involved in the interaction with DC, lead to inhibition of DC. APC, antigen presenting cell; CTLA4, cytotoxic T‐lymphocyte antigen 4; DC, dendritic cells; IDO, indoleamine 2,3‐dioxygenase; LAG‐3, lymphocyte activation gene 3; LAP, latency‐associated peptide; MHC‐II, major histocompatibility complex class II; PD‐1, programmed death‐1; PD‐L1, programmed death‐ligand 1; TGF‐β, tumor growth factor‐β; Treg, regulatory T cells.

CD25, also known as IL‐2 receptor is expressed at high levels on the surface of Treg cells. IL‐2 is an important signal that induces cell proliferation in vivo. Treg cells compete with effector cells for IL‐2 in the process of the immune response to prevent effector cells from acquiring a sufficient amount of IL‐2 to proliferate.^110^ Treg cells exert their functions through soluble intermediates. The extracellular and/or pericellular accumulation of adenosine causes an immunosuppressive response.^111^ CD39/CD73 expressed on Treg cells degrade adenosine triphosphate into adenosine, and the increase in the adenosine concentration in the microenvironment will inhibit antigen presentation by DCs.^112^ Factors, including granzyme‐A, granzyme‐B, perforin, anti‐inflammatory cytokines such as TGF‐β, IL‐10, and IL‐35 also play a pivotal role in the immune regulation of Treg cells (Figure 3).^113^, ^114^, ^115^

Cells that have high‐affinity receptors, express CTLA‐4, GITR, IL‐10, and TIGIT, in the contrary, cells with low‐affinity express more Epstein‐barr virus‐induced gene 3, which is responsible for inhibition of IL‐35, suggesting that affinity determines different inhibitory mechanisms.^116^

### 6.2 Treg therapy in RA

Numerous reports have been published on the regenerative function of Treg cells, and because an ideal therapeutic strategy would be to induce self‐tolerance before overt tissue damage occurs, researchers have increased the number of Treg cells. They have developed various strategies to restore Treg cell function. Reduction of Treg cells in vivo, including a reduction in the proinflammatory environment.^117^, ^118^, ^119^, ^120^, ^121^, ^122^, ^123^, ^124^, ^125^, ^126^ Specific Treg cell‐specific stimulators for gene expansion were used to promote Treg cell expansion or Treg cells were induced and expanded in vitro after the addition of immune complexes and then injected into patients.^127^ Adoptive transfer of Treg cells enhances survival in Scurfy mice, preventing from autoimmune diseases. Removing Treg cells before they become ill increases disease incidence and severity.^128^ At the same time, it was confirmed that the transfer of Treg cells could slow disease progression and these cells have the potential to treat autoimmune diseases.^128^, ^129^ Adoptive cell therapy (ACT) uses Treg cells isolated from blood based on their CD4 + CD25 + CD127− cell surface. Treg cells are expanded by treatment with anti‐CD3, anti‐CD28, and IL‐2 and then injected into the body.^130^ Expanded Treg cells were used to treat mouse autoimmune disease models before clinical use. Early studies were conducted in patients with type 1 diabetes, and graft‐versus‐host disease post‐bone marrow transplant and showed stable efficacy without serious side effects.^131^, ^132^, ^133^, ^134^, ^135^ The CIA, the animal model of RA also showed inhibition that markedly impeded the development of CIA.^136^

Importantly, when arthritis in these models was prevented, both T and B cells were suppressed by Treg cells, but also osteoclast bone destruction was directly suppressed, thereby preventing joint damage.^5^, ^137^

### 6.3 Limitations of Treg therapy

Before implementing ACT, some important technical issues need to be resolved. Since Treg cells recognize specific antigens, the first problem to be solved is how to isolate specific Treg cells in vitro. Both CD4 + CD127low/− and CD4 + CD127low/−CD25 + T cells were used for Treg expansion. Expansion of CD4 + CD127low/− cells needed the addition of rapamycin to maintain lineage purity. Expansion of CD4 + CD127low/−CD25 + T cells, especially the CD45RA+ subset, generates high yields of Treg cells that maintain high expression of Foxp3 expression even in the absence of rapamycin.^138^, ^139^ In the presence of anti‐CD3/anti‐CD28 and IL‐2, the number of cells elevates thousands‐folds with no loss of their inhibitory effect on Treg cells.^140^ IL‐2 is a key cytokine required for T cell activation and proliferation, and nTregs express high levels of CD25, receptors for IL‐2, making them highly sensitive to IL‐2 stimulation. IL‐2 (especially at low doses) preferentially promotes amplification of Treg cells.^141^ Low doses of IL‐2 directly increase the number of Treg cells in vivo, but this effect is short‐lived. Since the effect is greatly reduced when treatment is discontinued, it must be assumed that the effect of IL‐2 itself on other effector cells is also reduced. A second issue is how to effectively expand antigen‐specific Treg cells without losing their specificity or inhibitory function. Some studies have shown that expanded Treg cells tend to express IL‐17 and that CD4 + CD25 + Foxp3+ Tregs can transform into pathogenic Th17 cells upon repeated expansion.^142^, ^143^ These studies have shown that the epigenetic instability of Tregs. However, further studies showed that using CD45RA+ as an additional marker for Tregs isolation showed amplification‐induced epigenetic instability. It was shown minimizing the epigenetic instability associated with the increased inflammation associated with the conversion of Treg cells to Th17.^144^, ^145^

Adoptive therapy with Tregs has similar disadvantages to cell‐based therapies, such as the likelihood of becoming pathogenic cells. The absence of a specific antigen on the surface of Tregs also complicates the purification of Tregs and thus increases the risk of effector T contamination. The in vivo environment is complex and in vitro cell therapy is inevitably time‐consuming and expensive. As researchers have not conclusively determined whether in vivo expansion of Treg cells is superior to in vitro expansion, two approaches, such as applying autoantigen in incomplete adjuvant, have been proposed.^146^ Moreover, the combination of tolergenic dendritic cell (tolDC) and Treg‐inducing peptides to inactivate effector cells, promotes Treg cell function.^147^

## 7 Chimeric antigen receptor (CAR) T CELLS IN RA

### 7.1 CAR T cells and their effects

**Figure 4:**
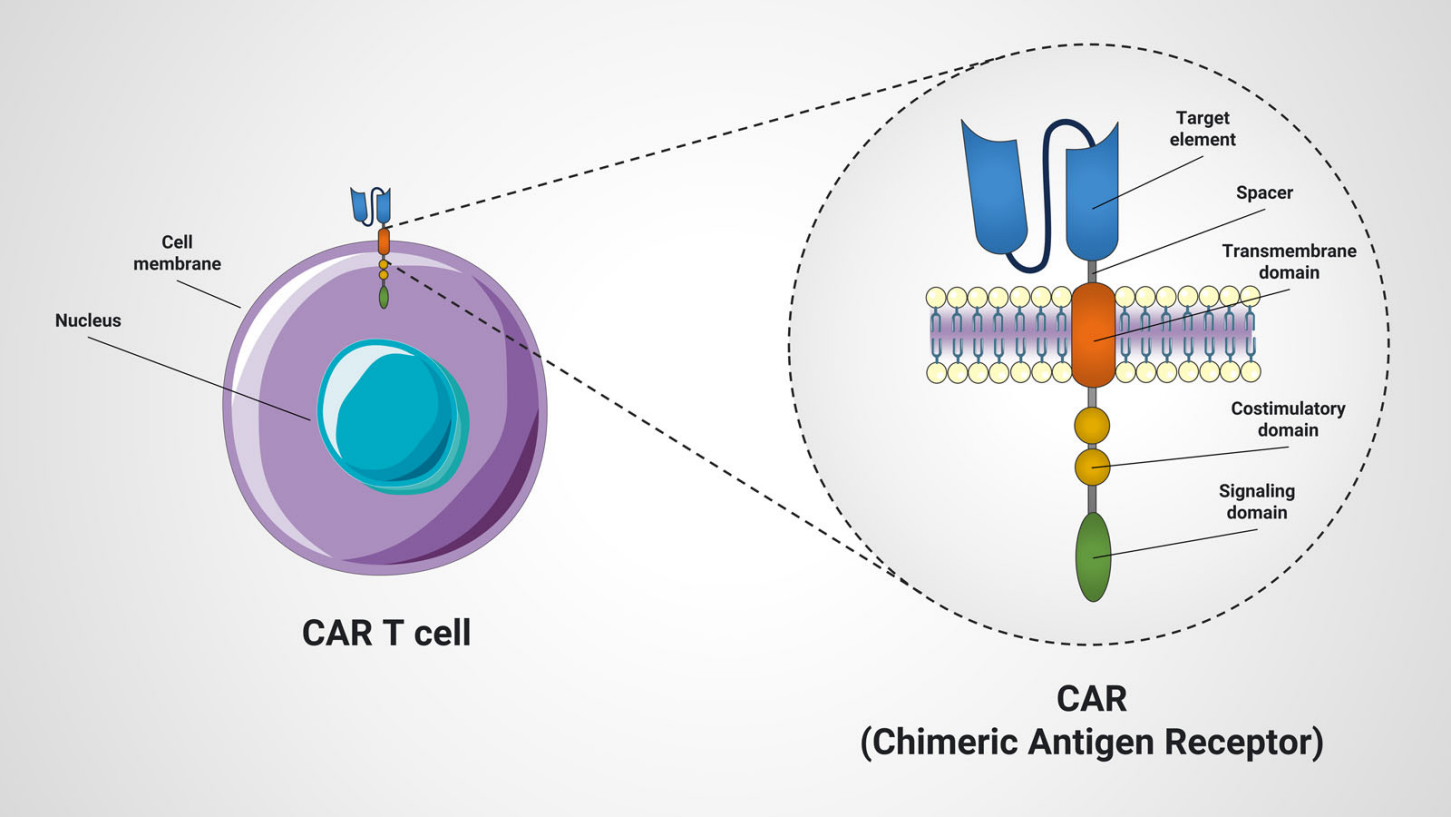
CAR structure. CARs are hybrid antigen receptors composed of ectodomain, transmembrane domain, and endodomain. Endodomain contains co‐stimulatory molecules domain as well as signaling domain. CAR, chimeric antigen receptor.

CARs are hybrid antigen receptors that redirect T cells to cells or tissues that express the antigen of interest, enabling T cells to recognize antigens independently of the MHC.^148^ A typical CAR consists of three major components, including an extracellular domain consisting of an antigen‐recognition domain, a hinge domain, an intracellular domain defined by a transmembrane domain and co‐stimulatory factors, and an intracellular signaling domain (Figure 4).^149^, ^150^

#### 7.1.1 Ectodomain

Ectodomains are domains of membrane proteins that are exposed outside of the cytoplasm and extracellular space and consist of single‐chain variable fragments (scFv) and spacers.^151^ The scFv serves as the signal peptide for the ectodomain within the CAR structure. It is formed from the variable fragment heavy chain and light chain of the monoclonal antibody (mAb), fused to a flexible linker.^152^ Antigen recognition domains are primarily derived from lymphocyte variable receptors, TCR mimetics, and mAbs. Among these, scFv are believed to be the most commonly selected antigen‐recognition domains for CAR construction.^153^ The hinge domain, also called spacer, creates a bridge‐like connection between the transmembrane domain and the antigen‐binding domain. Spacers, give the binding domains different ranges of motion and facilitate antigen recognition. Proteins used in the hinge region of CAR T cells are fragment crystallizable regions (Fc regions), antibody tail regions, IgG1, IgG4, IgD, and cell surface molecules such as CD28, CD8α, and CD7.^154^, ^155^

#### 7.1.2 Transmembrane domain

The transmembrane domain consists of a hydrophobic α‐helix that spans the membrane and provides a link between the extracellular and intracellular portions of the CAR molecule. This domain also affects the expression and stability of CAR.^156^, ^157^

#### 7.1.3 Endodomain

Although the first signal is provided by the fragment Fc receptor of the gamma or CD3ζ domain, full activation of T cells requires a second signal called co‐stimulation.^158^ Early T cells expressing first‐generation CARs (lacking co‐stimulatory domains) were deficient in cytokine secretion and had disappointing results in vivo.^159^, ^160^ As a result, first‐generation CARs were fused with co‐stimulatory molecules to enhance their proliferation and response. A variety of co‐stimulatory molecules have been studied, including CD28, 4‐1BB/CD137, OX40, ICOS, CD27, myeloid differentiation primary response 88/CD40, natural killer group 2 member D, CD244, and dNAX‐activating protein of 10 kDa. These molecules have been and will continue to be investigated in various clinical and preclinical studies. However, among these, CD28 and 4‐1BB/CD137 are the most commonly used co‐stimulatory factors for CAR‐T cell production.^161^, ^162^ Upon antigen recognition, CAR endodomains transmit activating and co‐stimulatory signals to T cells. Activation depends on the phosphorylation of immunoreceptor tyrosine‐based activation motifs present in the cytoplasmic domain of the TCR complex and the CD3 ζ domain.^163^, ^164^, ^165^

CAR‐T cells are bioengineered T lymphocytes that express specific receptors in their membranes and can recognize specific antigens on target cells without MHC restriction, thus interacting with target cells. CAR T cells are generated by isolating T cells from the patient's PB, inserting the CAR gene into the T cell genome, expanding the prepared CAR T cells, and reinjecting them into the patient.

Currently, CAR‐T cells are mainly used to treat various types of cancer. Recently, CAR‐T cells have also been used in preclinical studies in RA, colitis, systemic lupus erythematosus, pemphigus vulgaris, experimental autoimmune encephalomyelitis, and type 1 diabetes, offering new hope for treatment options in autoimmune diseases.^166^ Three different approaches were used in the preclinical models described above, including identifying specific antigens within target cells and inducing cytotoxic activity by CAR‐T cells to generate cytotoxic agents.

It acts on autoantibody‐releasing B cells via chimeric autoantibody receptor T cells (CAAR‐T) and binds to specific antigens in target cells to form CAR‐Treg on regulatory T lymphocytes. Treg exerts regulatory functions.^167^ CAAR‐T is a modified CAR variant that consists of a specific antigen, a transmembrane domain, and an intracellular signaling domain. CAAR‐T targets autoreactive B cells and exerts a selective cytotoxic function only on immune cells that transport receptors to specific autoimmune cells without universal immunosuppression.

When specific antigens on CAAR T cells are encountered and bound to specific autoantibodies expressed on B cells, the B cells are destroyed, thereby reducing autoantibody production^168^ CAR‐Treg regulates autoimmune T cells by inducing anergy and immunological ignorance. CAR‐Tregs produce immunosuppressive cytokines such as IL‐10, IL‐35, and TGF‐β, which induce apoptosis of effector T cells through Fas ligand, granzyme B/A, and perforin. CAR or CAAR targets only a single cell type, thus limiting the application of such novel therapeutic strategies in RA, where various types of autoreactive reactions occur. In one study, the first antigenic peptides for specific recognition by pathogenic B cells were developed and provided guidance for RA treatment according to a patient's specific autoantigen profile.^169^

### 7.2 CAR T cell therapy in RA

In RA, ACPA is one of the most specific serological markers associated with disease development. In an experiment, four citrullinated peptide epitopes, including citrullinated vimentin, citrullinated CTX‐II, citrullinated fibrinogen, tenascin‐C, and cyclocitrulline peptide‐1 were selected as ligands to target autoreactive B cells. Subsequently, engineered T cells expressing immobilized anti‐fluorescein isothiocyanate (FITC)‐CAR were constructed and tested for elimination of specific autoreactive B cells through recognition of the FITC‐tagged autoantigen peptide epitopes described above. The results showed that specifically redirected anti‐FITC CAR T cells could recognize the corresponding His FITC‐tagged citrullinated peptide epitopes and successfully lyse autoreactive B cell subsets of RA patients in vitro.

### 7.3 Limitations of CAR‐T cell therapy

Regarding CAR‐based therapy, an important obstacle in the design of CARs for autoimmune therapy is the relative scarcity of tissue‐specific antigens on the cell surface, because it is difficult to find suitable specific antigens to target the cells of interest in autoimmune diseases. Stability, durability, safety, effectiveness, manufacturing, and persistence must be confirmed for CAR‐T therapy to be applied in the clinic.^170^

## 8 tolDCs IN RA

### 8.1 tolDCs and RA

A protocol was developed by Hilkens and Isaacs to produce tolDC for the treatment of RA by pharmacological modulation of DC containing immunosuppressive agents, including dexamethasone (Dex), and 1,25 dihydroxyvitamin D3 (VitD3), and an agonist of Toll‐like receptor (TLR)‐4 (*Escherichia coli* lipopolysaccharide [LPS]).^171^ TolDC showed (1) higher expression of MHC class II in comparison with mature DC, (2) intermediate expression of co‐stimulatory molecules, including CD80 and CD86, and low expression of CD40 and CD83, (3) increase in anti‐inflammatory cytokine production, including high levels of IL‐10 and TGF‐β and low or undetectable levels of inflammatory cytokines, including IL‐12, IL‐23, and TNF‐α.^172^, ^173^ TLR‐4 agonists included in the tolDC generation protocol, have two reasons: (1) TLR‐4 activation is necessary for tolDC to process and present exogenous antigen on MHC class II.^173^ The similar protocol has been observed for immunogenic DC.^174^ Therefore, MHC‐II‐peptide complexes do not generate efficiently except that the tolDC receives a proinflammatory signal such as LPS during antigen uptake.^173^, ^174^ The tolDC capability to present antigens is crucial for the tolDCs success in the tolDC therapy, as the main aim of tolDC therapy is to induce T cell tolerance to the autoantigens (2) activation by TLR‐4 is also required for tolDC to acquire the capability for migrating in a C‐C chemokine receptor type 7 (CCR7)‐dependent manner, therefore, tolDC is enabled for migrating towards secondary lymphoid tissues, where they interact with T cells. Whether this migratory ability is required for the success of tolDC therapy in RA is not entirely obvious; however, evidence from the transplant setting shows that CCR7 expression by tolDC prolongs the survival of allografts in an animal model.^175^ These data demonstrate that secondary lymphoid tissues are crucial sites for the induction of immune tolerance,^176^ at least under normal, steady‐state conditions.

On the other hand, the situation may be different in RA, in which the joint tissues are infiltrated by T cells and APCs, including DC.^177^ Thus, it is likely that autoantigen presentation occurs in the joint. Therefore, it could be speculated that, in RA, tolDC would ideally have the capability to perform in several sites: in the diseased joints to anergize autoantigen‐specific effector T cells, and in the draining lymph node for induction of Tregs from autoantigen‐specific naive T cells. Notably, T cells from RA patients may be resistant to at least some tolerogenic signals; for example, they could resist IL‐10 and IDO.^178^ TolDC operates, at least partially, via TGF‐β and inhibits proliferation, and IFN‐γ production from PB T cells in vitro; however, whether tolDC can prevent autoreactive T cells in the rheumatoid joints remains to be determined.^171^

Though tolDC has a similar ability for processing and presentation of exogenous antigen as mature DC, tolDC has lower T cell stimulatory capacity than mature DC, in line with their lower expression of co‐stimulatory molecules and low production of proinflammatory cytokines.^172^ Moreover, tolDC induces hyporesponsiveness, “anergy,” in antigen‐experienced memory T cells, while polarizing naive T cells towards an anti‐inflammatory cytokine profile.^172^

Isaacs et al. have also shown that, in a mouse in vivo model of CIA, murine bone marrow‐derived tolDC generated with Dex, VitD3, and LPS have a therapeutic effect. Treatment of arthritic mice with tolDC (1 million cells injected intravenously three times over 8 days) significantly reduced the severity and progression of arthritis, whereas treatment with immunogenic mature DC exacerbated arthritis.^179^

TolDC showed therapeutic efficacy only when loaded with the immunizing antigen, CTX‐II. Treatment with tolDCs was associated with a decrease in Th17 cells and an increase in IL‐10‐producing T cells, as well as a decrease in proliferation of collagen‐specific type II T cells, possibly explaining its therapeutic effect. A clinical trial investigating autologous tolDC in RA is currently being performed on patients. This is a randomized, unblinded, placebo‐controlled, dose‐escalation Phase I study. Three dosing cohorts were designed, including 1 × 10^6^, 3 × 10^6^, and 10 × 10^6^ viable tolDC per patient. The main difference between this study and previous TolDC studies is the route of administration. TolDC will be administrated intra‐articularly, under arthroscopic guidance. Before administering, tolDC is administered into the joint, will be irrigated with saline. “Placebo” patients will also receive saline irrigation alone. Safety reasons are not the only reasons that tolDC is administered directly into the affected knee joint (if the joint flares up, it can be irrigated again, followed by an intra‐articular injection with corticosteroids), but it also allows the collection of synovial biopsies for the analysis of potential response biomarkers. Because TolDC targets diseased tissues, intra‐articular delivery may also have advantages compared to systemic delivery. In addition, tolDC may migrate to regional lymph nodes and deliver immunoregulatory signals necessary for the induction of immune tolerance. The first aim of this study was to evaluate the safety of intra‐articular injection of tolDC in RA patients. The secondary aim was to assess the tolerability/acceptability of the patient and the feasibility of TolDC treatment. This trial also had a number of exploratory aims, such as evaluating the effect of intra‐articular TolDC administration on RA disease activity (local and systemic). Potential response biomarkers in both synovial tissue and PB collected at multiple time points were also examined. Because the mechanisms underlying tolerance induction in vivo are still poorly understood, it is not possible to predict a comprehensive set of suitable biomarkers. Therefore, biomarker analysis uses a hypothesis‐free approach and includes analysis of leukocyte subsets using flow cytometry (DC subsets, T/B cell subsets, etc.), transcriptional profiling, and immunohistochemistry. The latter semiquantitatively assesses synovitis and synovial cell subsets. Evidence from the transplant field suggests that tolerance biomarkers are more likely to be found in synovial tissue than in PB, which may generate unexpected signals and require approaches such as transcriptional profiling. while it is planed to study pre‐ and posttreatment systemic autoreactivity; however, the uncertain nature of RA autoantigens makes this approach difficult.^171^, ^180^

A mouse model was used to address two major questions: (i) Is a maturation stimulus required for tolDC function in vitro and in vivo, and is maturation required for function in experimental arthritis (ii) Can TolDC modulate CD4+ T cell responses? To answer these questions, a comparison of mature and immature TolDC generated by Dex/VitD3 was performed in vitro. Thereafter, these TolDCs with naive or effector CD4+ T cells co‐treansfered and characterized the transferred T cells after 3 days using flow cytometry and Luminex multiplex. Furthermore, the suppressive capacity of tolDC in an experimental arthritis model was tested. It was found that TolDCs can modulate not only naive CD4+ T cell responses but also effector CD4+ T cells, as demonstrated by less proliferating and activating CD4+ T cells in vivo. In addition, Treg expansion (CD4 + CD25 + FoxP3+) was observed in the proliferating cell population in the presence of tolDC. Furthermore, it was shown that administered tolDC can suppress arthritis in a proteoglycan‐induced arthritis model. However, TolDCs require a maturation stimulus to exert this tolerance function in an inflammatory environment. These data are of great importance for optimizing TolDC therapy for future autoimmune diseases such as RA.^181^

### 8.2 Limitations

In addition to the challenges associated with the development and manufacturing of tolDC for clinical use, there are several challenges associated with the design of clinical trials. The timing of TolDC therapy is an important issue. In the case of transplantation, tolerogenic treatment can be applied before transplantation, which allows to create tolerance in an unprepared immune system. However, this is not the case in autoimmune disease, and tolDC is administered to patients with ongoing autoimmune disease who have already been diagnosed with a dysregulated autoimmune response. Especially in RA, the breakdown of tolerance to autoantigens can develop several years before the first symptoms of arthritis appear. It is generally believed that tolerogenic therapies, including tolDC therapy, have the greatest chance of success when applied early in the course of the disease.^182^ It is generally believed that tolerogenic therapies, including tolDC therapy, have the greatest chance of success when applied early in the course of the disease. Whether tolerogenic strategies can be successful in these settings remains to be seen, and the obvious risk is that further development of tolDC therapy may not occur if initial studies show little or no efficacy. Therefore measuring performance is a problem. The goal of TolDC therapy is to induce immune tolerance, but this can take time and may not lead to an immediate reduction in inflammation or other chronic symptoms. It was found that some immunomodulatory therapies that were ineffective in the short term appeared to be beneficial in the long term in RA patients.^183^ Therefore, both the timing of endpoints and the results to be measured must be carefully considered; Current RA clinical trial endpoints measure the consequences of inflammation, but this is unlikely to be an adequate marker for the short‐term “success” of tolDC therapy. There is an urgent need to develop suitable biomarkers for tolerance induction, which can then be used to monitor and target tolerogenic therapies such as tolDC. Collecting data on the expression of tolerance‐related genes and the functions of relevant immune groups before and after therapy is essential for the development of a robust and quantitative set of biomarkers. Such a series would allow for the measurement of short‐term therapeutic response in future studies of tolerogenic therapy and, if standardized, would allow comparison between different studies.

## 9 CONCLUSION

Taken together, these results suggest cell‐based therapy could improve the disease activity and severity in the animal models and human patients in RA. Cell‐based therapy resulted in an improvement in the clinical manifestation of the diseases, and skewed immunological parameters in favor of the disease improvement. Moreover, cell‐based therapy is safe, however, few complications have been reported. Engineered cells expressing various factors such as anti‐inflammatory cytokines or combination therapy utilizing stem cells and none‐stem cells and the other factors may be more effective in the modulation of immune responses and improvement of the disease. Despite the clear efficacy of cell‐based therapy in treatment of RA that is shown in many studies, some issues, including choice of the tissue that stem cells should be derived, autologous versus allogenic or xenogenic origin of cells, type of stem cell such as MSC or HSC, predictive factors of the disease remain greatly unknown and should be addressed in the next studies to increase therapeutic efficacy of cells therapies, including stem cell and none stem cells and minimize the complications of the transplantation.

## AUTHOR CONTRIBUTIONS

Maryam Zare Moghaddam and Somayeh Ghotloo devised the project outlines, and performed literature reviewing and data processing. Maryam Zare Moghaddam wrote the manuscript under the critical revision of Somayeh Ghotloo. Mohammad Javad Mousavi designed the figures and revised the manuscript.

## CONFLICT OF INTEREST STATEMENT

The authors declare no conflict of interest.
